# PRAD-Hybrid CNN (PRADHC): A Deep Learning Model for Assisted Diagnosis of Prostate Cancer on MRI

**DOI:** 10.2174/0115734056413897251202130301

**Published:** 2026-03-09

**Authors:** Jingpeng Liu, Lingxuan Hou, Yang Xu, Yong Zhang, Huantao Zong

**Affiliations:** 1 Department of Urology, Beijing Tiantan Hospital, Capital Medical University, Beijing, China; 2 College of Biomedical Engineering, Sichuan University, Chengdu, Sichuan, China; 3 Department of Nuclear Medicine, Beijing Chaoyang Hospital, Capital Medical University, Beijing, China

**Keywords:** Deep learning, Prostate cancer, Precise assisted diagnosis, Convolutional neural networks, Magnetic resonance imaging, Early-stage cancers

## Abstract

**Background:**

Prostate cancer is a prevalent malignancy in males, with prostate MRI imaging as the primary diagnostic method. However, this method is subjective and can miss early-stage cancers, necessitating more efficient diagnostic techniques.

**Method:**

In this study, we introduced the PRAD-Hybrid CNN (Prostate Adenocarcinoma Hybrid Convolutional Neural Network, PRADHC) model, a novel amalgamation of EfficientNet and Residual Blocks, which was developed and validated on 1,528 MRI images from 64 patients. By strategically increasing the number of Convolutional Neural Network (CNN) layers in the EfficientNet architecture, our model improved the diagnostic accuracy inherent to the original EfficientNet. Additionally, the integration of Residual Networks (ResNet) successfully mitigated the gradient vanishing issue often encountered during the training of deeper models, thereby significantly enhancing training accuracy. This innovative model, thus, offers clinicians an efficacious tool for assisted diagnosis.

**Result:**

The PRADHC model, upon validation, achieved an accuracy of 99.34% and an AUC of 99.34%, a 4% improvement over the conventional EfficientNet. The baseline elementary CNN model achieved 95.72% accuracy and 96.74% AUC, which are still lower than the PRADHC model.

**Discussion:**

The superior performance of PRADHC can be attributed to the synergistic integration of EfficientNet’s multi-scale feature extraction and residual learning, which facilitates deeper network optimization without degradation. Compared with single-architecture CNN models, the hybrid design enhances robustness to MRI appearance variability and improves discrimination between malignant and non-significant prostate tissue.

**Conclusion:**

This study introduces a novel deep learning model specifically designed for automated prostate cancer diagnosis. This model aims to enhance diagnostic accuracy, especially in the early stages of the disease. Such advancements have the potential to enhance the diagnostic proficiency of both radiologists and urologists, enabling more informed treatment planning. However, it is imperative to acknowledge that false-positive lesion detections remain a limitation of AI-assisted diagnostic tools. Nevertheless, this system can serve as a valuable supplementary instrument for radiologists in their diagnostic endeavors.

## 1 INTRODUCTION

Prostate cancer (PCa) stands as the world's second most commoncancer accordingto global statistics, with North and South America, Europe, Australia, and the Caribbean as the regions with the highest incidence rates [1, 2]. The risk of this disease arisesfrom multiple factors, including age, family medical history, genetic factors, and eating patterns. The development of this condition has been linked to external factors, which include tobacco use, physical exercise, particular drugs, and workplace dangers [1, 3]. The worldwide numbers of new prostate cancer cases and deaths will rise to 2.3 million because of changing population demographics, which include increasing numbers of people and an aging population [4].

The main cancer type affecting men remains prostate cancer, but this disease tends to progress slowly, which results in good survival outcomes when detected at an initial stage [5]. The current methods for prostate cancer detection face difficulties in identifying prostate cancer at its earliest stages with high accuracy, which makes surveillance systems and early detection methods essential for achieving better treatment results.

The high mortality from prostate cancer is largely due to patients being diagnosed at advanced stages, which makes treatment ineffective [6, 7]. The diagnostic tools for prostate cancer include prostate biopsies, PSA assays, digital rectal examinations, MRI, and general health screenings [6]. PSA screening has become widespread throughout the 21^st^ century, which has proven essential for reducing the number of PCa cases [8]. The PSA test shows specific results about prostate gland health, but it does not provide information about prostate cancer. The PSA levels, which rise above normal ranges, do not provide sufficient diagnostic accuracy because they can result from non-cancerous conditions, such as BPH and prostatitis [6, 9]. The gold standard for PCa diagnosis is biopsy, but these procedures have two major drawbacks: they are invasive, and they produce false-negative test results [10]. Research has made progress in prostate cancer detection and treatment through the discovery of new molecular markers, the development of mpMRI and PSMA-PET scans as advanced imaging technologies [6, 11]. The medical community now supports MRI scans as the first diagnostic test, which should be performed before prostate biopsy procedures for patients who have high-risk conditions [12]. The rapid development of artificial intelligence (AI) and machine learning technologies requires an autonomous algorithm to detect prostate cancer at its earliest stages, as early detection enables better medical outcomes through early intervention.

In recent years, with rapid advances in artificial intelligence and deep learning, there has been an increasing focus on deep learning for the diagnosis and detection of prostate cancer. Recent breakthroughs have demonstrated remarkable improvements in diagnostic accuracy, with Islam *et* *al*. achieving 99.64% accuracy using ResNet50 with transfer learning [13] and Talaat *et al*. reporting 97.40% sensitivity with modified ResNet50 architectures and dual optimizer strategies [14]. For instance, Abbasi *et al*. conducted a comparative study on the performance of various Convolutional Neural Networks (CNN) models in transfer learning applications [15]. Furthermore, they evaluated the efficacy of distinct machine learning methodologies when employed as classifiers. Their findings revealed an optimal combination of transfer learning models, laying a foundational groundwork for transfer learning-based prostate cancer detection. This has the potential to serve as a benchmark for radiologists in detecting prostate cancer. The integration of attention mechanisms has emerged as a particularly promising approach. Duran *et al*. developed ProstAttention-Net [16], a dual-branch attention model that achieved an 87.5% Dice score for prostate segmentation, significantly outperforming traditional U-Net architectures. Similarly, Saha *et al*. demonstrated that 3D CNNs incorporating attention mechanisms with clinical priors substantially reduced false-positive rates in bi-parametric MRI analysis [17], achieving superior lesion-level detection accuracy. Liu *et al*. [18], leveraging deep learning and CNNs, trained a model using 10,056 magnetic resonance imaging images, achieving an accuracy rate of 78.15%. The methodologies proposed in their research carry significant clinical implications and can be widely adopted for the grading and staging of prostate cancer and other oncological tasks. Zheng *et al*. introduced a novel deep learning architecture based on convolutional neural networks [19], named XmasNet, designed to classify prostate cancer lesions. By training XmasNet end-to-end and augmenting data with three- dimensional rotations and slices, they incorporated lesion 3D information, achieving an AUC of 84%. de Vente *et al*. also introduced a neural network architecture capable of detecting and grading prostate cancer in an end-to-end manner [20]. This innovative approach offers a clinically meaningful prostate cancer detection system that transcends the capabilities of conventional classification methods. This showcases the immense potential deep learning holds in addressing such challenges. Nonetheless, there exist challenges and limitations in diagnosing prostate cancer using deep learning. For example, due to model constraints, there is room for improvement in diagnostic accuracy. Overly complex models can suffer from limited generalization capabilities, while overly simplified ones might not achieve high accuracy. The absence of a standardized preprocessing method poses substantial challenges in real-world clinical applications. Moreover, there is currently a lack of extensive databases with a broad demographic coverage. The limited diversity in training samples is a significant hindrance to the performance of deep learning models. Recent multi-center validation studies have begun to address these challenges, with comprehensive analyses showing that properly validated hybrid architectures can maintain high performance across diverse institutional datasets [21, 22].

The current study establishes an automatic prostate diagnostic system that uses MRI data to produce precise diagnostic results to support medical staff during diagnostic work. The system enables us to reduce the time radiologists spend on image analysis, helping them manage their work better and remove related obstacles. The research presents the PRAD-Hybrid CNN, a model that combines EfficientNet functionality with Residual Block architecture for prostate adenocarcinoma detection. The system maintains EfficientNet's top diagnostic accuracy for prostate conditions on MRI while addressing the gradient vanishing problem that deep models experience during training. This method produces enhanced predictive results and better overall performance. The EfficientNet architecture adjusts its width, depth, and resolution to achieve maximum efficiency and accuracy when processing an MRI image. The integrated system enables our team to perform fast, precise prostate cancer detection using MRI image processing. The research workflow of our study appears in Fig. (**1**).

## 2 METHODS

### 2.1 Data Sources

In this study, we leveraged the Transverse Plane Prostate Dataset from TCIA as our primary source for both training and validation data [23]. This dataset was comprised of a collection of 1528 transverse prostate MRI images from 64 distinct patients. Each patient in the database has undergone a singular prostate MRI scan. During dataset compilation, all images were already converted from DICOM to JPEG. Ultimately, the dataset was split into two distinct subsets using a holdout method: 30% of the images were allocated to validation, with the remaining 70% to training. Furthermore, we categorized the images into two groups, 'significant' and 'not significant', and split them into training (70%) and validation (30%) sets. Representative images from both 'significant' and 'not significant' categories within the database are shown in Fig. (**2**).

Early-stage prostate cancer often presents symptomatically similar to benign prostatic hyperplasia (BPH) or may manifest no distinct symptoms at all. Primary screening for such cases usually involves a digital rectal examination (DRE) or testing for prostate-specific antigen (PSA) levels. Notwithstanding these methods, definitive diagnosis of prostate cancer predominantly necessitates histopathological analyses of biopsy specimens or tissues obtained from transurethral resection of the prostate. Magnetic Resonance Imaging (MRI) serves as a vital diagnostic modality, elucidating the integrity of the prostate capsule, potential invasion of neighboring tissues or organs by the tumor, as well as revealing pelvic lymph node involvement and bone metastases. This aids significantly in clinical staging.

Multiparameter MRI (mpMRI) stands out as superior to other imaging modalities when diagnosing prostate cancer. The Prostate Imaging Reporting and Data System (PI-RADS) provides a standardized method for prostate cancer localization, diagnosis, and risk assessment through 1.5T or 3.0T multi-parameter MRI scans. The classification system includes T2-weighted imaging (T2WI) as its main application within PI-RADS. The T2WI imaging technique shows the prostate structure in detail, enabling doctors to detect prostate gland abnormalities and assess seminal vesicle invasion, extracapsular extension, and lymph node involvement. The peripheral zone contains tumors that cause noticeable clinical symptoms because they appear as hypointense signals on T2WI images. The test shows a restricted ability to identify particular conditions. The T2WI imaging technique shows hypointense regions that can be caused by several benign conditions, including prostatitis, hemorrhage, glandular atrophy, benign hyperplasia, post-biopsy scars, and post-therapeutic changes.

### 2.2 Data Preprocessing

The proposed model required a complete set of preprocessing methods, which we used to achieve its optimal performance. The MRI images received spatial filtering and histogram normalization, resulting in better predictive performance. We randomly divided the images into a 7:3 training-to-validation ratio for model development and evaluation.

Moreover, the database inherently showed a significant imbalance, with the number of pathological MRI images vastly outnumbering those of normal MRI images. To rectify this, we adopted various data augmentation techniques to balance the dataset. Techniques, such as flipping, cropping, stretching, and noise addition, were employed. These not only bolstered the model's resilience against irregular input but also, by balancing the dataset, substantially improved its overall performance.

### 2.3 Model Building

#### 2.3.1 Proposed Model

Currently, numerous research endeavors utilizing deep learning for the automatic detection of prostate cancer are emerging. The most successful recent approaches have employed hybrid architectures that combine the strengths of different neural network designs. Xu *et al*. demonstrated that multimodal fusion approaches integrating T2W, DCE, and DWI sequences achieved robust diagnostic performance [21], while Sherafatmandjoo *et al*. reported that combining multiple ResNet50 networks with clinical data significantly improved accuracy to 96% [22]. Hassan *et al*. combined several deep learning methodologies to introduce a novel auto-classification algorithm [24]. This algorithm is adept at detecting prostate cancer from ultrasound (US) and magnetic resonance imaging (MRI) scans. Their optimal model reported an impressive accuracy of 97% on the US images test set and 80% on MRI images, suggesting potential for deployment in intelligent clinics or hospitals for effective prostate cancer detection and interpretation. Zhao *et al*. developed a deep learning model based on bi-parametric MRI (bpMRI) from multiple centers [25], tailored for diagnosing clinically significant prostate cancer, achieving an accuracy of 83.5%. Furthermore, Li *et al*. devised a deep learning model [26], leveraging mpMRI combined with whole-slide histopathological data to enhance prostate cancer diagnostic capabilities, resulting in an AUC of 87.1%. However, the diagnostic performance of these models was suboptimal with low accuracy, rendering them unsuitable for direct deployment in hospital systems. Given this backdrop, we introduced a highly precise model for prostate cancer diagnosis named PRADHC.

PRADHC is developed by integrating Residual Blocks into EfficientNet. Introduced by Google Research's Mingxing Tan and Quoc V. Le in 2019 [27], EfficientNet's primary philosophy revolves around a balanced expansion of the network's width, depth, and resolution, aiming for enhanced efficiency. This architecture has proven particularly effective in medical imaging applications, with recent studies demonstrating its superiority in feature extraction and computational efficiency for prostate cancer detection [13, 14]. It employs a module, termed MBConv, rooted in MobileNetV2, coupled with residual connections and squeeze-and-excitation modules. Moreover, these networks are optimized *via* AutoML and neural architecture search, thereby demonstrating commendable performance across multiple tasks. Residual Blocks, proposed by He *et al*. in 2015 [28], are designed to train the so-called “residual function” by directly adding the input to the output, referred to as “skip connections” or “shortcut connections”. This facilitates efficient gradient propagation throughout the network, enabling the construction of exceptionally deep neural architectures that excel across various tasks. After several experiments, our model's optimal training was identified to be 15 epochs, ensuring peak performance without overfitting. The optimal learning rate and batch size were determined to be 0.0001 and 64, respectively.

The MBConv structure in EfficientNet, together with ResBlock, enables the model to extract features at different scales, resulting in better prostate cancer detection accuracy. Adding residual connections to EfficientNet prevents gradient vanishing or explosion, which becomes more likely as networks become deeper. The system allows researchers to build complex neural networks that detect multiple complex patterns in images, achieving better results in prostate cancer identification. The model becomes more robust through its built-in adaptive data augmentation methods, which EfficientNet provides alongside its ResBlock residual structure. The system demonstrates high resistance to both noisy and complex prostate MRI data due to its design. The structure of the PRADHC model is shown in Fig. (**3**).

#### 2.3.2 Contrast Model

In this study, to thoroughly evaluate the performance of the PRADHC model, we constructed a variety of baseline models under the same training and validation framework for detailed comparative analysis, including EfficientNet, CNN, ResNet, MobileNet, and NasNet. Among them, CNN, MobileNet, and NasNet served as performance benchmarks representing different architectural characteristics, while EfficientNet and ResNet were used to analyze the performance distinctions intrinsic to their respective architectures.

The concept of CNN dates back to the 1980s, but its revolutionary breakthrough in deep learning and computer vision applications commenced with Yann LeCun's research in 1989 [29]. The classical CNN consists of input layers, convolutional layers, non-linear activation functions, pooling layers, fully connected layers, and output layers, proving its efficiency in computer vision tasks, such as image recognition and object detection. Utilizing such a foundational model for comparative performance accentuates the superior applicability and innovative aspects of the PRADHC model. Similarly, researchers, such as Soni *et al*., introduced an SEMRCNN model based on a CNN [30] that autonomously extracts prostate cancer lesions from multi-parametric MRI (MP-MRI) and achieves precise lesion segmentation.

MobileNet was first proposed by Google researchers, including Howard, in 2017 [31]. Its innovation lies in adopting depth-wise separable convolutions in place of traditional convolutions, aiming to reduce computational costs and model size. Furthermore, it offers flexibility in modulating model size and performance through two hyperparameters: the width multiplier and the resolution multiplier. Such innovative strategies ensure MobileNet's commendable performance while substantially reducing computational overhead.

The NasNet model was developed by the Google Brain team in 2017 [32]. It employs Neural Architecture Search (NAS) to automatically identify the optimal network architecture. Contrary to traditional methodologies, NasNet's design emphasizes optimizing the repetitive modules or “cells” within the network rather than the entire network structure, by conducting the optimization search through an RNN controller. This approach effectively streamlines the search space, enhancing the modularity and reusability of the architecture, marking a significant stride in automated machine learning (AutoML) within the computer vision domain.

### 2.4 Experiment

In this study, the training and validation datasets for all models were randomly partitioned in a 7:3 ratio. To ensure optimal parameter tuning, gradient-based methods were employed, iteratively refining all parameters within both the PRADHC model and the baseline models to their optimal states. Moreover, by appropriately adjusting the training epochs, we ensured that all models converged without overfitting. All experiments were conducted on a Windows 11 Professional Edition system, using Python 3.10.9. Packages, such as TensorFlow 2.9, Scikit-learn, Scikit 0.0.post1, SciPy 1.10.0, and Matplotlib, were used to support model architecture development and results validation. The hardware configuration for our experiments comprised an Intel Core i7 10750H CPU (with a base frequency of 2.6GHz, a maximum turbo frequency of 5GHz, and structured as six cores/twelve threads) alongside an NVIDIA GeForce GTX 1080Ti GPU (boasting an 8GB memory size and a memory bus width of 128bit) (**Supplementary material**).

### 2.5 Model Evaluation

In this study, to provide a more in-depth and objective evaluation of the performance disparities among various models, we adopted accuracy (ACC), recall (REC), precision (PRE), F1-score (F1), and the ROC curve along with its area under the curve (AUC) as the assessment metrics. To compute these metrics with precision, True Positives (TP), True Negatives (TN), False Positives (FP), and False Negatives (FN) were meticulously recorded and incorporated into the corresponding formulas. The associated confusion matrix is depicted in Fig. (**4**).

ACC represents the proportion of samples correctly predicted by the model relative to the total number of samples. It is a fundamental metric for evaluating a model's predictive capability. The formula for ACC is given by:

**Table:** 

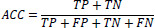	(1)

PRE measures the proportion of samples that were predicted as positive and were actually positive, relative to all samples predicted as positive. Its formula is:

**Table:** 

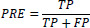	(2)

REC indicates the fraction of actual positive samples that were correctly predicted as positive. It is calculated using the following formula:

**Table:** 

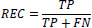	(3)

The F1-score is the harmonic mean of precision (PRE) and recall (REC), serving as a comprehensive metric. It is given by the following equation:

**Table:** 

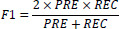	(4)

The ROC curve delineates the relationship between the true positive rate (TPR) and the false positive rate (FPR) at various decision thresholds. The x-axis of the ROC curve represents the FPR, while the y-axis denotes the TPR. Ideally, the ROC curve should approach the upper-left corner, signifying a high TPR and low FPR. The AUC quantifies the area beneath the ROC curve, providing a standardized measure of the model's overall classification performance. AUC values range between 0 and 1, where 1 denotes a perfect classifier, and 0.5 is equivalent to random guessing. Generally, a higher AUC suggests superior classifier performance. All statistical analyses and performance metrics (including ACC, PRE, REC, F1, and AUC) were calculated using Python libraries, such as Scikit-learn (v1.3) and SciPy (v1.10.0).

## 3 RESULTS

### 3.1 Model Comparison Result

After rigorous experimentation and training, this study meticulously fine-tuned the model parameters to achieve optimal performance. To objectively compare the performance across various models, the number of training epochs was consistently set to 15 based on repeated experimental results. For the PRADHC model, we configured the learning rate at 0.0001 and the batch size to 64. This configuration successfully optimized the model's performance without any signs of overfitting. The training iteration curve for the PRADHC model is depicted in Fig. (**5a**).

To visually represent the model's performance, we calculated various metrics for the PRADHC model in comparison to the baseline models. The PRADHC model yielded an ACC, PRE, REC, F1, and AUC of 99.34%, 99.31%, 99.30%, 99.30%, and 99.34%, respectively. All these metrics significantly outperformed those of the baseline models. This demonstrates the superior performance of the PRADHC model in predicting prostate cancer *via* prostate MRI. Additionally, we plotted the ROC curves and confusion matrices for all models to offer a more intuitive insight into their performance, as shown in Fig. (**5b**).

Among the baseline models, EfficientNet achieved the best performance, with ACC, PRE, REC, F1, and AUC scores of 95.74%, 97.04%, 93.67%, 95.27%, and 95.57%, respectively (Table **1**). All these metrics were inferior to those of the PRADHC model. Notably, our proposed PRADHC model was derived from both EfficientNet and ResidualNet architectures. To provide a more comprehensive assessment, we also evaluated other baseline models, excluding these two.

Among these, the basic CNN model emerged as the top performer with an ACC of 95.72%, marginally trailing behind EfficientNet. This underscores the exceptional capability of the PRADHC model in predicting prostate cancer using prostate MRI.

### 3.2 Model Visualization Results

A convolutional neural network generates feature maps as its output at each processing stage. The feature maps show how the image processing system responds at various levels of its hierarchical structure. The first few layers of the network detect basic features, which include edge detection and texture identification. The higher levels of the system contain abstract concepts that might represent different parts of objects. The model reveals its main attention areas through visual inspection of its feature maps. In line with this understanding and to offer a clearer perspective on the training efficacy, we extracted the activation maps from the final convolutional layer of the PRADHC model to enhance its interpretability, as depicted in Fig. (**6**). The activation map delineates the regions of focal attention during the feature extraction process of the PRADHC model. Significantly, the model adeptly identifies the prostate region and subsequently classifies it. The classification process becomes more understandable when we consider that convolutional networks perform feature extraction through hierarchical structural pattern detection, starting with basic elements and progressing to more complex ones. The PRADHC model demonstrates its ability to identify essential prostate anatomical features, which enables it to achieve strong performance in prostate structure identification and classification tasks.

## 4 DISCUSSION

The research presents PRADHC as a deep learning system that uses prostate MRI data to achieve an exact prostate cancer diagnosis. The PRADHC model combines the EfficientNet architecture with the Residual Block architecture through its complex design structure. The MBConv structure from EfficientNet enables the model to extract features at different scales. The Residual Block structure integration helps address the gradient vanishing problem that occurs during EfficientNet training, thereby improving both model performance and training efficiency. The architectural combination unites the best elements of both original models to create a system that will improve the accuracy of prostate cancer diagnosis. The PRADHC model achieved outstanding results in prostate cancer prediction through MRI analysis, surpassing baseline models after a thorough assessment. The experimental results show that our model performs at a level comparable to those of multiple well-known architectures presented in recent studies. Islam *et al*. [13] achieved comparable results with 99.64% accuracy using transfer learning approaches, while Hamm *et al*. [33] demonstrated that explainable AI models can achieve an AUC of 0.89 with the added benefit of interpretability. The system achieved consistent high performance across different hybrid architectures by using EfficientNet with Residual Blocks [14, 17, 22]. The model achieved exceptional results, with 99.34% accuracy (ACC) and an AUC of 99.34%. The performance results showed that the new models achieved better results than the original baseline models.

Our initial testing revealed that EfficientNet achieved good results at the beginning of training, but its performance did not improve as we continued training. Additionally, the model faced the notorious gradient vanishing issue. The solution to these problems involved adding Residual Block functionality to the EfficientNet framework. As a result, the proposed PRADHC model outperformed the conventional EfficientNet. The MBConv architecture in EfficientNet focused on processing multiple scales of input data because this approach is essential for prostate cancer diagnosis using MRI. The developed system provides an effective method for automated prostate cancer prediction using prostate MRI [34].

Magnetic Resonance Imaging (MRI) has become an indispensable tool in the diagnostic regimen for suspected prostate malignancies. Prolonged analysis of these images not only places a significant cognitive burden on radiologists but can also lead to mental fatigue, risking potential lapses in their attention. Hence, the advancements in the PRAD-Hybrid CNN (PRADHC) model stand paramount. Machine learning, a subset of artificial intelligence, focuses on enabling systems to learn from large datasets by applying probabilistic and statistical methods. This, in turn, empowers these systems to make informed decisions or prognoses on novel data. In medical imaging, the fusion of feature engineering and machine learning, through computer-aided detection and diagnosis (CAD), has demonstrated its potential to enhance radiological accuracy. Recent clinical validation studies have confirmed this potential, with Cai *et al*. demonstrating fully automated systems that match the performance of experienced radiologists [35] and Karagoz *et al*. reporting that anatomically guided networks maintain robust performance across multi- institutional datasets [36]. These advances underscore the readiness of AI systems for clinical integration, particularly when employing hybrid architectures that balance accuracy with computational efficiency [16, 21, 33]. Such systems have the capability not only to enhance diagnostic precision but also to reduce the associated time and financial costs.

However, this study does possess certain limitations. The data originated from a single public cohort (64 patients; 1,528 transverse MRI slices); although we used patient-wise splitting and five-fold cross-validation to reduce leakage and split sensitivity, external multi-centre validation is needed to establish generalisability. Images were 2D JPEG slices converted from clinical DICOM, lacking volumetric and multi- sequence context that may contain subtle signal and spatial continuity. The present work focuses on image-level binary classification and does not evaluate lesion-level localisation/grading (*e.g*., PI-RADS or Gleason) or case-level aggregation across slices; interpretability was limited to qualitative saliency/activation maps. Finally, we did not report standardised deployment metrics (*e.g*., hardware-specific latency) or component-wise ablation. These assessments will be addressed in future studies using larger, multi-institutional datasets.

**Fig. (6):**
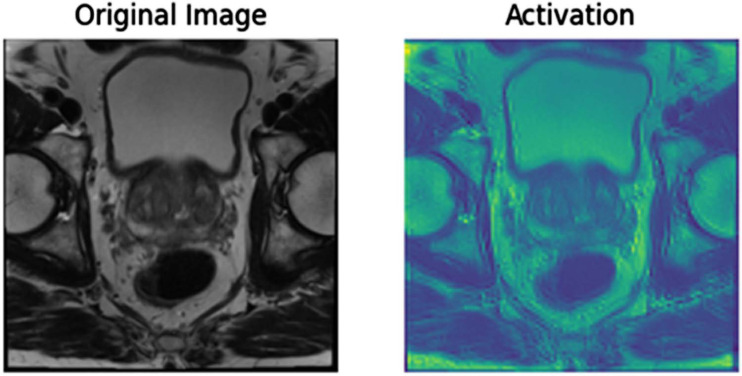
The activation mapping of the PRADHC model.

Clinically, PRADHC is best used as a second reader that flags suspicious slices, not as an autonomous decision-maker, and, as an image-level binary model, its outputs should be integrated with radiologist judgement and clinical context. Activation maps aid interpretability but are qualitative; therefore, prospective reader studies and external validation are needed before deployment, consistent with the principle that AI assists, not replaces, clinicians (Fig. **6**).

## CONCLUSION

This study introduces a novel deep learning model, named PRAD-Hybrid CNN, tailored for the automatic diagnosis of prostate cancer. The architecture synthesizes the strengths of EfficientNet and the Residual Block. Such a fusion not only harnesses the superior performance of EfficientNet but also leverages the Residual Block to mitigate overfitting issues often encountered in deeply layered models. The outcome is an enhanced capability for precise identification of prostate cancer. The PRAD-Hybrid CNN seeks to elevate the diagnostic quality associated with early-stage prostate cancer, delivering rapid, autonomous, precise, and efficient diagnoses. Its application stands to enhance the proficiency of both radiologists and urologists in diagnosing and planning treatment for prostate cancer. While grappling with false- positive lesion identifications remains an inherent challenge for AI-assisted detection algorithms, the PRAD-Hybrid CNN can be envisioned as a complementary tool in a radiologist's arsenal. Future endeavors should pivot towards advancing this model for seamless integration into clinical practice.

## Figures and Tables

**Fig. (1):**
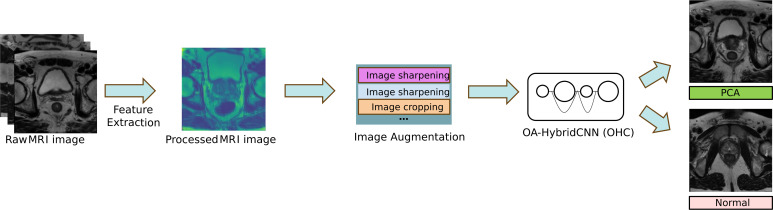
The workflow of this study.

**Fig. (2):**
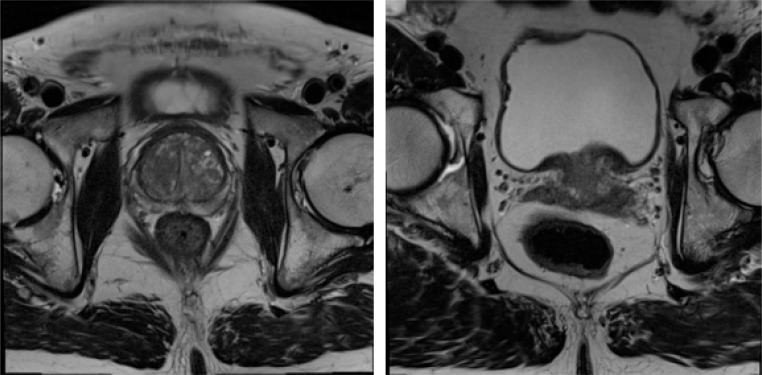
Transverse plane images of prostate from the dataset (the left one is a normal prostate’s MRI image, and the right one is an MRI image from a prostate cancer patient).

**Fig. (3):**
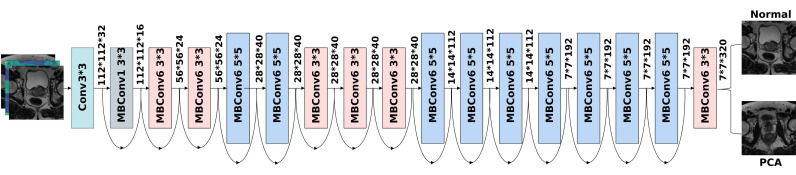
The structure of the PRADHC model.

**Fig. (4):**
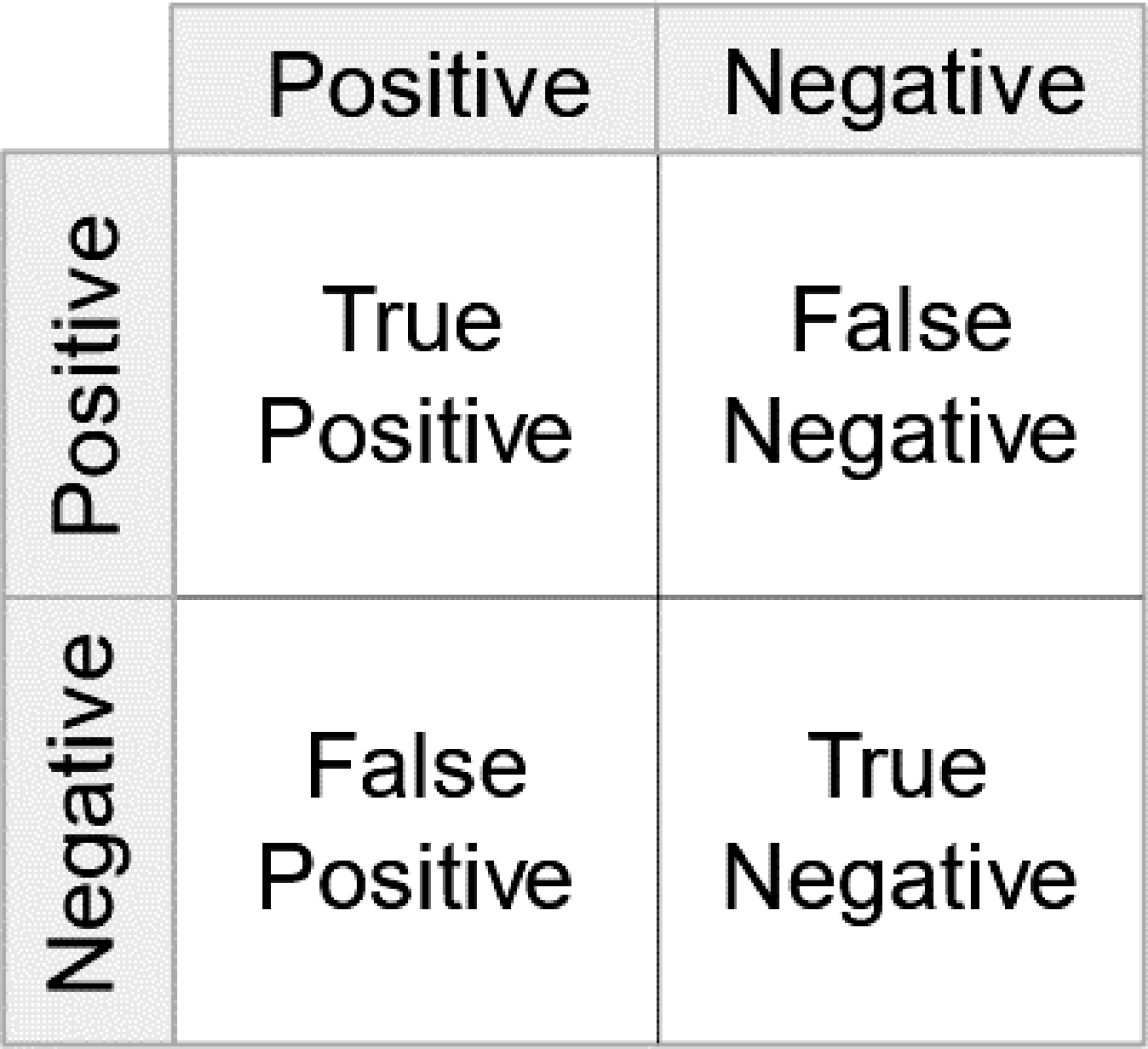
The confusion matrix of evaluation.

**Fig. (5):**
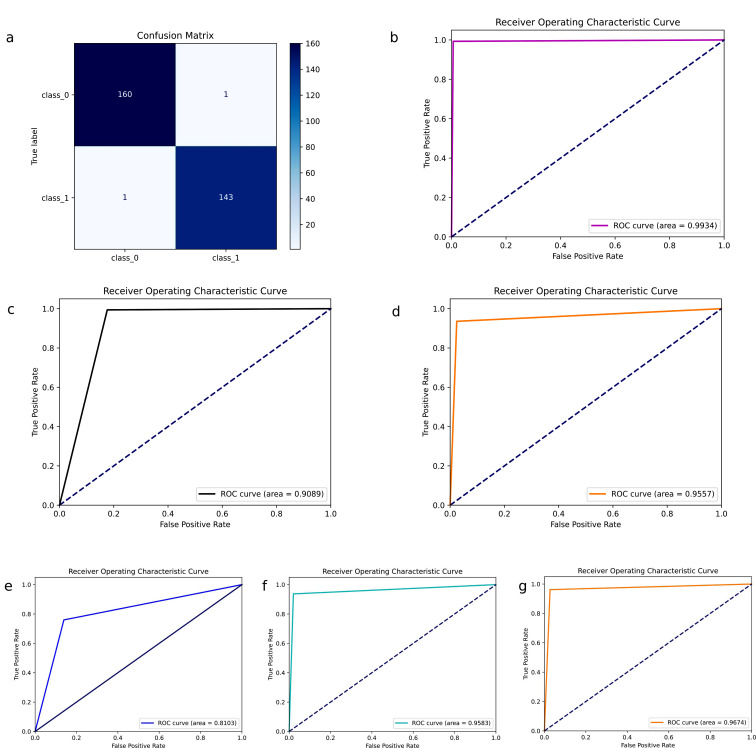
(**a**) The training flow of the PRADHC model. (**b**) The ROC curve and confusion matrix of the PRADHC model. (**c**-**g**) The ROC curves of ResNet, EfficientNet, NasNet, MobileNet, and CNN.

**Table 1:** Model performance of the proposed model and baseline models.

	**ACC**	**PRE**	**REC**	**F1**	**AUC**
**New**	**99.34%**	**99.31%**	**99.30%**	**99.30%**	**99.34%**
EfficientNet	95.74%	97.04%	93.67%	95.27%	95.57%
CNN	95.72%	96.20%	97.44%	96.82%	96.74%
ResNet	91.48%	86.63%	99.39%	92.57%	90.89%
MobileNet	94.10%	96.45%	91.28%	93.79%	95.83%
NasNet	80.98%	77.84%	86.09%	81.76%	81.03%

## Data Availability

The dataset used in this study originates from the publicly accessible ProstateX Challenge collection hosted on The Cancer Imaging Archive (TCIA). The dataset can be accessed at: https://www.cancerimagingarchive.net/collection/prostatex/. All code used for data preprocessing, model development, training, and evaluation is provided as supplementary attachments to this submission.

## LIST OF ABBREVIATIONS

MRI: Magnetic Resonance Imaging
BPH: Benign Prostatic Hyperplasia
AI: Artificial Intelligence
PSMA-PET: Prostate-Specific Membrane Antigen Positron Emission Tomography
mpMRI: Multiparametric Magnetic Resonance Imaging
DRE: Digital Rectal Examination
PSA: Prostate-Specific Antigen
