# Supplementary material for: PRAD-Hybrid CNN (PRADHC): A Deep Learning Model for Assisted Diagnosis of Prostate Cancer on MRI
Source: Curr Med Imaging. 2026 Mar 9;22:e15734056413897. doi: 10.2174/0115734056413897251202130301 (PMC13458438; doi:10.2174/0115734056413897251202130301)
Supplement: Supplementary file 1 [file CMIM-22-E15734056413897_SD1.pdf]

1875-6603/26 *Send Orders for Reprints to reprints@benthamscience.net*

BENTHAM SCIENCE

CrossMark

Current Medical Imaging

Content list available at: https://benthamscience.com/journals/cmir

Supplementary Material

PRAD-Hybrid CNN (PRADHC): A Deep Learning Model for Assisted Diagnosis of Prostate Cancer on MRI

Jingpeng Liu[1], Lingxuan Hou[2], Yang Xu[3], Yong Zhang[1,*] and Huantao Zong[1,*]

[1]*Department of Urology, Beijing Tiantan Hospital, Capital Medical University, Beijing, China*
[2]*College of Biomedical Engineering, Sichuan University, Chengdu, Sichuan, China*
[3]*Department of Nuclear Medicine, Beijing Chaoyang Hospital, Capital Medical University, Beijing, China*

PRAD

```
import os
from tensorflow.keras.optimizers import Adam
import pandas as pd
from tensorflow.keras.callbacks import ModelCheckpoint
import seaborn as sns
import matplotlib.pyplot as plt
import tensorflow as tf
from numpy import ndarray, dtype, void
from sklearn.model_selection import train_test_split
from tensorflow.keras.preprocessing.image import ImageDataGenerator
from tensorflow.keras.models import Sequential
from tensorflow.keras.layers import Conv2D, concatenate, AveragePooling2D, Dense, Dropout, GlobalAveragePooling2D, Input, BatchNormalization, Activation, Add, Multiply
from tensorflow.keras.models import Model
from tensorflow.keras.layers import DepthwiseConv2D, Reshape
from tensorflow.keras.applications import ResNet50, Xception, InceptionV3, MobileNetV2, VGG16, DenseNet121
from tensorflow.keras.applications import EfficientNetB0
from tensorflow.keras.layers import (
GlobalAveragePooling2D, Dense, Dropout, Conv2D, BatchNormalization,
ReLU, Add, DepthwiseConv2D, Input
)
from tensorflow.keras.models import Model
from tensorflow.keras.optimizers import Adam
train_dir = 'change to your dir'
test_dir = 'change to your dir'
val_dir = 'change to your dir'
# Define the categories
categories = ["0", "1", "2"] #classes

train_data = []
for category in categories:
folder_path = os.path.join(train_dir, category)
images = os.listdir(folder_path)
count = len(images)
train_data.append(pd.DataFrame({"Image": images, "Category": [category] * count, "Count": [count] * count}))
train_df = pd.concat(train_data, ignore_index=True)
gpus = tf.config.experimental.list_physical_devices('GPU')
if gpus:
try:
for gpu in gpus:
tf.config.experimental.set_memory_growth(gpu, True)
except RuntimeError as e:
print(e)
```

DOI: 10.2174/0115734056413897251202130301, 2026, *22*, e15734056413897

```
# Set the image size
image_size = (512, 512)
# Set the batch size for training
batch_size = 8

# Set the number of epochs for training
epochs = 10
# Data augmentation and preprocessing
train_datagen = ImageDataGenerator(
rescale=1./255,
rotation_range=20,
width_shift_range=0.1,
height_shift_range=0.1,
shear_range=0.1,
zoom_range=0.1,
horizontal_flip=True,
vertical_flip=True,
fill_mode="nearest"
)
train_generator = train_datagen.flow_from_directory(
train_dir,
target_size=image_size,
batch_size=batch_size,
class_mode="categorical"
)
test_datagen = ImageDataGenerator(rescale=1./255)
test_generator = test_datagen.flow_from_directory(
test_dir,
target_size=image_size,
batch_size=batch_size,
class_mode="categorical",
shuffle=False
)
val_datagen = ImageDataGenerator(rescale=1./255)
val_generator = val_datagen.flow_from_directory(
val_dir,
target_size=image_size,
batch_size=batch_size,
class_mode="categorical",
shuffle=False
)

def ResBlock(x, filters, kernel_size=3, stride=1):
shortcut = x
x = Conv2D(filters, kernel_size, strides=stride, padding='same')(x)
x = BatchNormalization()(x)
x = ReLU()(x)

x = Conv2D(filters, kernel_size, strides=1, padding='same')(x)
x = BatchNormalization()(x)

if stride != 1 or shortcut.shape[-1] != filters:
shortcut = Conv2D(filters, 1, strides=stride, padding='same')(shortcut)
shortcut = BatchNormalization()(shortcut)

x = Add()([x, shortcut])
x = ReLU()(x)
return x

def MBConv(x, in_channels, out_channels, expansion=6, stride=1):
shortcut = x
hidden_dim = in_channels * expansion

# 1x1 pointwise conv (expansion)
x = Conv2D(hidden_dim, 1, padding='same')(x)
x = BatchNormalization()(x)
x = ReLU()(x)

# Depthwise convolution
x = DepthwiseConv2D(3, strides=stride, padding='same')(x)
x = BatchNormalization()(x)
x = ReLU()(x)

# 1x1 pointwise conv (projection)
x = Conv2D(out_channels, 1, padding='same')(x)
x = BatchNormalization()(x)

# Residual connection
if stride == 1 and in_channels == out_channels:
x = Add()([x, shortcut])
return x

image_size = (512, 512)
base_model = EfficientNetB0(weights=None, include_top=False,
```

```
input_shape=(image_size[0], image_size[1], 3))
x = base_model.output
x = ResBlock(x, filters=256)
x = MBConv(x, in_channels=256, out_channels=256, expansion=6, stride=1)
x = GlobalAveragePooling2D()(x)
x = Dense(512, activation='relu')(x)
x = Dropout(0.5)(x)
predictions = Dense(len(categories), activation='softmax')(x)
model = Model(inputs=base_model.input, outputs=predictions)
learning_rate = 0.00001
optimizer = Adam(learning_rate=learning_rate)
model.compile(optimizer=optimizer, loss="categorical_crossentropy", metrics=["accuracy"])
history = model.fit(
train_generator,
steps_per_epoch=train_generator.samples // batch_size,
epochs=epochs,
validation_data=val_generator,
validation_steps=val_generator.samples // batch_size
)
fig, ax1 = plt.subplots(figsize=(10, 5))
ax1.plot(history.history['accuracy'], color='k', label='Train Accuracy')
ax1.plot(history.history['val_accuracy'], color='g', label='Validation Accuracy')
ax1.set_xlabel('Epoch')
ax1.set_ylabel('Accuracy', color='k')
ax1.tick_params('y', colors='k')
ax2 = ax1.twinx()
ax2.plot(history.history['loss'], color='k', linestyle='--', label='Train Loss')
ax2.plot(history.history['val_loss'], color='m', linestyle='--', label='Validation Loss')
ax2.set_ylabel('Loss', color='k')
ax2.tick_params('y', colors='k')
lines, labels = ax1.get_legend_handles_labels()
lines2, labels2 = ax2.get_legend_handles_labels()
ax2.legend(lines + lines2, labels + labels2, loc=0)
plt.title('Model Accuracy and Loss')
plt.show()
loss, accuracy = model.evaluate(test_generator, steps=test_generator.samples // batch_size)
print("Test Loss:", loss)
print("Test Accuracy:", accuracy)
```

COMPARE_MODELS

```
import os
from tensorflow.keras.optimizers import Adam
import pandas as pd
import seaborn as sns
import matplotlib.pyplot as plt
import tensorflow as tf
from numpy import ndarray, dtype, void
from sklearn.model_selection import train_test_split
from tensorflow.keras.preprocessing.image import ImageDataGenerator
from tensorflow.keras.models import Sequential
from tensorflow.keras.layers import Conv2D, concatenate, AveragePooling2D, Dense, Dropout, GlobalAveragePooling2D, Input, BatchNormalization, Activation, Add
from tensorflow.keras.models import Model
from tensorflow.keras.layers import DepthwiseConv2D
from tensorflow.keras.applications import ResNet50, Xception, InceptionV3, MobileNetV2, VGG16, DenseNet121
train_dir = 'change to your dir'
test_dir = 'change to your dir'
val_dir = 'change to your dir'
# Define the categories
categories = ["0", "1", "2"] #classes
# Load and preprocess the dataset
train_data = []
for category in categories:
folder_path = os.path.join(train_dir, category)
images = os.listdir(folder_path)
count = len(images)
train_data.append(pd.DataFrame({"Image": images, "Category": [category] * count, "Count": [count] * count}))
train_df = pd.concat(train_data, ignore_index=True)
gpus = tf.config.experimental.list_physical_devices('GPU')
if gpus:
try:
for gpu in gpus:
tf.config.experimental.set_memory_growth(gpu, True)
```

```
except RuntimeError as e:
print(e)
# Set the image size
image_size = (512, 512)
# Set the batch size for training
batch_size = 8

# Set the number of epochs for training
epochs = 50
# Data augmentation and preprocessing
train_datagen = ImageDataGenerator(
rescale=1./255,
rotation_range=20,
width_shift_range=0.1,
height_shift_range=0.1,
shear_range=0.1,
zoom_range=0.1,
horizontal_flip=True,
vertical_flip=True,
fill_mode="nearest"
)
train_generator = train_datagen.flow_from_directory(
train_dir,
target_size=image_size,
batch_size=batch_size,
class_mode="categorical"
)
test_datagen = ImageDataGenerator(rescale=1./255)
test_generator = test_datagen.flow_from_directory(
test_dir,
target_size=image_size,
batch_size=batch_size,
class_mode="categorical",
shuffle=False
)
val_datagen = ImageDataGenerator(rescale=1./255)
val_generator = val_datagen.flow_from_directory(
val_dir,
target_size=image_size,
batch_size=batch_size,
class_mode="categorical",
shuffle=False
)
# you can change to desire models
base_model = Xception(weights='imagenet', include_top=False, input_shape=(image_size[0], image_size[1], 3))
model_name = 'Xception'
x = base_model.output
x = GlobalAveragePooling2D()(x)
x = Dense(512, activation='relu')(x)
x = Dropout(0.5)(x)
predictions = Dense(len(categories), activation='softmax')(x)
model = Model(inputs=base_model.input, outputs=predictions)
learning_rate = 0.00001
optimizer = Adam(learning_rate=learning_rate)
model.compile(optimizer=optimizer, loss="categorical_crossentropy", metrics=["accuracy"])
history = model.fit(
train_generator,
steps_per_epoch=train_generator.samples // batch_size,
epochs=epochs,
validation_data=val_generator,
validation_steps=val_generator.samples // batch_size
)
plt.figure(figsize=(10, 6))
line_styles = ['-', '--', '-.', ':']
plt.plot(history.history['accuracy'], color='blue', linestyle=line_styles[0], markevery=1, label='Training Accuracy')
plt.plot(history.history['val_accuracy'], color='orange', linestyle=line_styles[1], markevery=1, label='Validation Accuracy')
plt.plot(history.history['loss'], color='green', linestyle=line_styles[2], markevery=1, label='Training Loss')
plt.plot(history.history['val_loss'], color='red', linestyle=line_styles[3], markevery=1, label='Validation Loss')
plt.title('Training and Validation Accuracy and Loss', fontsize=16)
plt.xlabel('Epoch', fontsize=14)
```

```
plt.ylabel('Value', fontsize=14)
plt.legend(loc='best', fontsize=12)
plt.grid(True)
plt.tight_layout()
plt.savefig(f'D:\\work\\lung multi\\OA-PIC\\{model_name}_combined_flow.png', dpi=300)
plt.show()
# Evaluate the model
import numpy as np
import tensorflow as tf
import seaborn as sns
import matplotlib.pyplot as plt
loss, accuracy = model.evaluate(test_generator, steps=test_generator.samples // batch_size)
print("Test Loss:", loss)
print("Test Accuracy:", accuracy)
```

© 2026 The Author(s). Published by Bentham Science Publisher.

This is an open access article distributed under the terms of the Creative Commons Attribution 4.0 International Public License (CC-BY 4.0), a copy of which is available at: https://creativecommons.org/licenses/by/4.0/legalcode. This license permits unrestricted use, distribution, and reproduction in any medium, provided the original author and source are credited.

HOW TO CITE:
Liu J, Hou L, Xu Y, Zhang Y, Zong H. PRAD-Hybrid CNN (PRADHC): A Deep Learning Model for Assisted Diagnosis of Prostate Cancer on MRI. Curr Med Imaging, 2026; 22: e15734056413897.
http://dx.doi.org/10.2174/0115734056413897251202130301
